# Early onset sepsis: clinical observation or risk factors approach?

**DOI:** 10.1016/j.jped.2024.10.007

**Published:** 2024-11-30

**Authors:** Sofia Romay Oliveira, Cintia Lopes, Alana Beatriz Coelho Basilio

**Affiliations:** Universidade Federal do Paraná (UFPR), Complexo Hospital de Clínicas, Unidade Materno Infantil – Alojamento Conjunto e Centro Cirúrgico Obstétrico, Curitiba, Paraná, Brazil

**Keywords:** Early-onset sepsis, Newborn, Streptococcal infections, Risk factors, Blood count, C-reactive protein

## Abstract

**Objectives:**

To compare the perinatal risk factors approach for early-onset sepsis (EOS), which is based on categorical risk stratification, with the clinical observation-based approach, evaluating their impact on laboratory testing frequency, the use of antibiotic therapy, and EOS incidence.

**Methods:**

Retrospective observational study, conducted from November 2021 to March 2022. Newborns (NB) at 34 wk of age were included and clinical data from prenatal care, birth, hospitalization, and laboratory tests were evaluated.

**Results:**

Sample of 1,086 newborns. Ninety-seven NB (8.9 %) underwent infectious screening in the clinical observation approach versus 279 (26.5 %) in the perinatal risk factors approach, which represents a 65.2 % decrease in the clinical observation approach (*p* < 0.01). Under the perinatal risk factors approach, 35 (3.2 %) of NBs received empirical antibiotic therapy for EOS, versus only 22 (2.0 %) in the clinical observation approach, which would be a 37.1 % decrease in the clinical observation strategy (*p* < 0.01). We found no difference in the incidence of culture-confirmed EOS.

**Conclusion:**

The clinical observation approach, when compared to the perinatal risk factors approach, reduces laboratory testing and the use of antibiotic therapy, with no impact on the incidence of EOS. Further research is required to determine the best way to systematize serial examinations of NB's and which symptoms would be the best predictors of EOS.

## Introduction

Early-onset sepsis (EOS) is the systemic infection of the newborn (NB) through vertical transmission in the first days of life. An infection is classified as EOS when it occurs within the first 72^1^ hours of the NB's life, or up to 6 days after birth if it is caused by Group B Streptococci (GBS).^2^ The golden standard for diagnosis is the identification of a microbiological agent in the culture of an otherwise sterile body fluid, but cases of culture-negative EOS have been described.^1^^,^^3^^,^^4^ Countries with well-structured health systems have an EOS incidence of 0.5 to 0.98 per thousand live births.^1^^,^^4^ Mortality is influenced by gestational age, birth weight, and etiological agent, ranging from 2 % to 50 % of confirmed cases.^1^^,^^4^

Given the seriousness of the disease, universal screening of pregnant women for GBS colonization and the use of intrapartum prophylactic antibiotic therapy have been recommended since 2002. These have been crucial measures for reducing the incidence of EOS in the last few decades.^2^^,^^4^ Since 2010, neonatal management has also been recommended for secondary prevention of EOS, based on perinatal risk factors. According to the NB's risk classification, this approach employs infectious screening with laboratory tests or prophylactic antibiotic therapy.^5^

Currently, there are three possible approaches to assess NBs at risk of EOS. The perinatal risk factors approach involves a high rate of laboratory tests and prophylactic antibiotic therapy in NBs at low risk of EOS. The other two are the multifactorial risk assessment approach, which uses the Neonatal Sepsis Calculator, and the clinical observation approach,^1^ both of which have a lower frequency of laboratory testing and antibiotic therapy with no negative outcomes in terms of incidence and mortality due to EOS.^6^, ^7^, ^8^

Considering the different forms of neonatal management for EOS prevention, this study aims to compare the perinatal risk factors approach with the clinical observation approach, evaluating their impact on the frequency of laboratory testing, the use of antibiotic therapy, and EOS incidence.

## Methods

We conducted an analytical, observational, and cross-sectional study with retrospective data collection, from November 11, 2021, to March 15, 2022, at a tertiary-care teaching hospital integrating the Brazilian public health system which registers approximately 3600 live births per year. NBs have their vital signs checked by nurses every six hours in the rooming house and every three hours in the neonatal intensive care unit (ICU), and are examined by pediatricians at least once a day in the rooming house and twice a day in the ICU.

Live newborns with gestational age ≥ 34 wk were included in the study. Patients with genetic syndromes, severe malformations, neonatal asphyxia, and NBs under seven days of life who were transferred to another hospital were excluded. The following data was collected from maternal and newborn electronic medical records: gestational age; maternal GBS colonization; indication of intrapartum antibiotic prophylaxis, and whether it was adequate (one dose at least four hours before delivery); duration of membrane rupture; premature rupture of ovarian membranes; maternal intrapartum temperature; newborn symptoms, when present; collection of tests and their indications; use of antibiotic therapy; and whether there was readmission up to the seventh day of life for those who were discharged before then.

The approach used at CHC-UFPR is based on maternal and neonatal risk factors, as shown in Table 1.

**Table 1:** Perinatal risk factors for EOS.

NB of a mother with a previous child with GBS EOS
GBS-colonized mother and labor or membrane rupture, with inadequate intrapartum antibiotic therapy^a^
Unknown GBS colonization and one of the following, with inadequate intrapartum antibiotic therapy^a^:
Premature membrane rupture
Membrane rupture over 18 h
Premature labor
Mother with intrapartum fever
Membrane rupture over 18 h (or for an uncertain duration), regardless of GBS colonization
Clinical urinary tract infection or positive urine culture, current or previous without negative control urine culture
Mother with signs of infection: fever, foul-smelling amniotic fluid or other indications of chorioamnionitis

NB, newborn; GBS, Group B streptococcus; EOS, early-onset sepsis.

a At CHC-UFPR, adequate intrapartum antibiotic prophylaxis requires two doses of an adequate antibiotic (usually, ampicillin) ministered before delivery — which is different from what was considered adequate in this study.

The NB was considered to have undergone infectious screening when C-reactive protein (CRP) and blood count were drawn, whether it was associated with blood culture (or other cultures) or not. The researchers only considered cases where the NB received ampicillin or crystalline penicillin combined with gentamicin as prophylactic antibiotic therapy for EOS.

The entire sample was subjected to the perinatal risk factor-based approach, which consists of screening patients with risk factors for EOS, as well as those with associated symptoms. For the retrospective study, the sample was hypothetically subjected to the clinical observation-based approach, which consists of collecting laboratory tests solely from patients with symptoms suggestive of EOS that are not explained by any other etiology.

When the collection for laboratory tests did not meet the risk factors in Table 1 and the NB did not have symptoms associated with EOS, the approach was classified as a "questionable approach".

The following EOS classifications were considered: "no sepsis" for NBs with no symptoms and negative culture; "confirmed EOS" for NBs with clinical symptoms suggestive of EOS up to seven days of life, which were not explained by any other etiology, and with positive culture; "clinical EOS" for NBs with symptoms but no growth in cultures; and "asymptomatic bacteremia" for NBs who had positive blood culture for the pathogen but no symptoms. The classification "laboratory alterations" was also created to identify NBs who received antibiotics due to abnormal infectious screening tests, even in the absence of clinical symptoms. However, for analytical purposes, the latter group was eventually classified as "without sepsis".

The data were collected and tabulated in Microsoft Excel® spreadsheets and analyzed using the Statistical Package for the Social Science - SPSS® software (IBM® SPSS® Statistics v. 25.0, SPSS Inc, Chicago, USA). The results were expressed as means, medians, minimum values, maximum values, and standard deviations (quantitative variables), or as frequencies and percentages (qualitative variables). The chi-squared test was used for inferential analysis; p-values of <0.05 were considered significant. A comparison was made between the two approaches in terms of three outcomes: test collection, antibiotic administration, and the incidence of EOS.

The study was approved by the Research Ethics Committee of the CHC-UFPR.

## Results

During the study period, a total of 1126 live births were included, with 29 NBs excluded due to meeting certain exclusion criteria. Eleven medical records were incomplete and were thus considered lost samples. Therefore, the study sample consisted of 1086 NBs. The risk factors for EOS in the analyzed sample are displayed in Table 2.

**Table 2:** Risk factors for early-onset sepsis in the sample.

		n	%
Gestational age	≥ 37 wk	979	90.1
	34 to 36+6 wk	107	9.9
GBS	Negative	616	56.7
	Positive	155	14.3
	Unknown	315	29.0
IAP indication	No	870	80.1
	Yes	213	19.6
	Insufficient data	2	0.2
Received IAP (1 dose ≥ 4 h after delivery)	No	145	13.4
	Yes	98	9.0
	Insufficient data	32	3.0
Membrane rupture	In full	301	27.7
	Prolonged membrane rupture ≥ 18 h	85	7.8
	Route < 18 h	445	41.0
	Uncertain time	14	1.3
	Route in the act	241	22.2
Premature membrane rupture	No	958	88.2
	Yes	107	9.9
	Unknown	21	1.9
Maternal intrapartum fever (axillary temperature ≥ 37.8 °C)	No	378	34.8
	Yes	4	0.4
	Unknown	704	64.8

IAP, Intrapartum Antibiotic Prophylaxis; GBS, Group B Streptococcus. <, less than; ≥, greater than or equal to.

Of the 1086 newborns, 383 underwent infectious screening: 279 were singled out by the perinatal risk factors approach (182 for risk factors alone and 97 for symptoms) and 104 for reasons considered questionable. The clinical observation approach showed a 65.2 % decrease in infectious screenings when compared to the perinatal risk factors approach (*p* < 0.01). Only five NB's had cerebrospinal fluid (CSF) collected for culture analysis, and all were negative.

Under the perinatal risk factors approach, 3.2 % (35) of the total sample received empirical antibiotic therapy for EOS after infectious screening due to risk factors. Of these 35 NBs who received empirical antibiotic therapy, 37.1 % (13) were tested only due to risk factors and received antibiotic therapy exclusively for laboratory alterations, while the other 62.9 % (22) underwent infectious screening and antibiotic therapy for being symptomatic.

Of the 104 NBs that underwent infectious screening for reasons considered questionable, with no risk factor and no symptoms, six (5.7 %) received empirical antibiotic therapy due exclusively to laboratory alterations.

Under the clinical observation approach, only the 22 (2.0 %) NBs who presented symptoms would have received antibiotic therapy. When comparing the approaches, there would be a 37.1 % decrease in antibiotic therapy use in the clinical observation strategy (*p* < 0.01). Table 3 shows the frequency of symptoms and risk factors amongst this population.

**Table 3:** Presence of risk factors for EOS and clinical symptoms amongst those who received antibiotic therapy.

		Presence of risk factors (n, %^a^)		Total
		No	Yes	
EOS clinical symptoms	No	0 (0 %)	19 (46.3 %)	19
	Yes	11 (26.8 %)	11 (26.8 %)	22
Total		11	30	41

EOS, early-onset sepsis.

a Total sample percentage.

There were no cases of confirmed EOS or asymptomatic bacteremia. Of the 437 blood cultures, six were positive, but all of them contained agents considered contaminants according to a Technical Note from the Brazilian National Health Surveillance Agency (ANVISA).^9^

Of the 22 (2.0 %) symptomatic NBs who received antibiotic therapy, 5 (22.7 %) were initially classified as having clinical EOS, but had antibiotic therapy suspended within 72 h and were classified as EOS-free. Seventeen NBs were classified as having clinical EOS, resulting in a prevalence of 15.6 cases per thousand live births. Of the 19 (1.74 %) NBs who received antibiotics but did not have clinical EOS, 16 (84.2 %) had laboratory alterations only; 3 (15.8 %) received empirical antibiotics due to risk factors but had antibiotic therapy suspended within 72 h and were also classified as EOS-free.

Table 4 summarizes the main findings of this study.

**Table 4:** Comparison of approaches to assessing newborns at risk of EOS.

	Perinatal risk stratification approach	Clinical observation approach	Decrease	Value of *P*
Infectious screening sampling	279	97	−65.2 %	< 0.01
Antibiotic therapy	35	22	−37.1 %	< 0.01
Clinical EOS	17	17	0 %	< 0.01

EOS, early-onset sepsis.

There were no readmissions within seven days of life due to EOS and no deaths related to EOS in the analyzed period.

## Discussion

This study aimed to determine whether the clinical observation approach, compared to the perinatal risk factors approach, could yield certain benefits, such as a decrease in laboratory tests and antibiotic therapy, without any adverse effects related to EOS. We observed a decrease of 65.2 % in laboratory tests and 37.1 % in the use of antibiotic therapy (*p* < 0.01) in the clinical observation approach, with no significant impact on the number of EOS cases.

Several publications corroborate the findings of this study. Cantoni et al*.*, in a prospective study of 15,239 full-term NBs, compared an approach based on clinical observation and laboratory tests with an approach based on clinical examination alone, and found a 91 % decrease in the frequency of laboratory testing and a 58 % decrease in antibiotic therapy, but no difference in EOS incidence.^8^ Berardi et al.,^10^ Castellanos et al*.*^11^ and Vatne et al.^12^ also compared the clinical observation approach to the perinatal risk factors approach: all found a decrease in laboratory testing and, in the former two, a decrease in the use of antibiotic therapy, without worse EOS outcomes.

Joshi et al. evaluated 227 asymptomatic children of mothers with chorioamnionitis who underwent the clinical observation approach and observed a decrease of 82.7 % in laboratory testing and 88.4 % in antibiotic therapy, with no confirmed cases of EOS.^13^ The same approach was used by Frymoyer et al. to evaluate all NB's with a gestational age over 35 wk: they observed a 59 % decrease in laboratory testing and a 63 % decrease in antibiotic therapy.^7^

Schmitt et al*.* evaluated the latest French protocol, which is based on clinical observation. They found a 95 % decrease in laboratory testing but no difference in the number of infections, hospitalizations, or mortality.^14^ Ramón et al. compared 3 strategies: perinatal risk factors, Neonatal Sepsis Calculator, and clinical observation. They applied the calculator and clinical observation approaches retrospectively and hypothetically, and reported a possible decrease in laboratory testing and antibiotic use when both approaches were used.^15^

The blood count and CRP approach has limited diagnostic value for asymptomatic NBs. Hornik et al. evaluated 160,092 NBs with suspected EOS and concluded that no blood count parameter has the sensitivity to identify an NB with EOS.^16^ The study by Hofer et al. demonstrated that CRP has low sensitivity at the onset of symptoms, as it takes 24 to 48 h to reach a serum peak.^17^ In a 2021 review, Puopulo et al. reinforced that blood count and CRP should not be used to determine antibiotic therapy and that the patient's clinical and blood culture results should guide the procedures.^18^

Besides the questionable usefulness of infectious screening, it is known that venipunctures themselves carry inherent risks. NBs have a lower pain threshold than pediatric and adult populations and exposure to painful procedures during this period may be related to alterations in pain regulation pathways, delayed growth, and inadequate neuropsychomotor development.^19^^,^^20^

Another benefit of the clinical observation approach is the decrease in the number of NBs exposed to the effects of antibiotic therapy in the neonatal period. Possible side effects of this kind of treatment include an increased risk of developing asthma, food allergies, inflammatory bowel diseases, and obesity, as well as an impact on breastfeeding due to the separation of the mother-baby binomial.^1^^,^^21^, ^22^, ^23^, ^24^ Additionally, the clinical observation approach has also shown an economic advantage by decreasing antibiotic therapy and hospital length of stay.^25^

The risk of NBs with good general condition presenting EOS is low.^26^^,^^27^ In a study with children of mothers colonized by GBS with inadequate prophylaxis, the researchers observed that no asymptomatic NBs had a positive blood culture and that all NBs with a positive blood culture for GBS presented symptoms.^28^ Furthermore, the majority of culture-positive EOS cases are symptomatic in the first few hours of life, requiring a course of action before the time when an infectious screening is usually carried out.^7^^,^^8^^,^^12^^,^^29^^,^^30^ Moreover, the decrease in laboratory tests does not delay the start of antibiotic therapy in NBs suspected of having EOS.^8^^,^^29^ The symptom-based approach can even reduce the time to start antibiotic therapy.^12^

Once it has been established that the clinical observation approach is safe, there are still gaps regarding how frequently the serial physical examination should be performed. The frequency of NB assessment in this study is lower when compared to other publications. Berardi et al., for example*,* compared protocols for clinical observation approaches, and the frequency of NB assessing varied from six to ten times in the first 24 h of life, compared to five times in this study.^26^

The clinical EOS prevalence of 15.6 per thousand live births is higher than that reported in other studies. However, this comparison is limited by the lack of consensus on the concept of EOS. Most studies consider that a positive culture is imperative to define the diagnosis of EOS.^1^^,^^3^^,^^31^^,^^32^ A systematic review from 2023 evaluated concepts of neonatal sepsis and concluded that there is significant variation in definitions, making it necessary to establish an international consensus and thus allowing better analysis of the results found in the literature.^3^

The present study has some limitations: it is a retrospective observational study and, due to the low incidence of EOS, the study sample size is small. No confirmed EOS cases were identified, which limited the opportunities to better analyze the performance of each approach. The pediatrician who attended the NBs was responsible for defining the assessment of EOS symptoms, the decision to perform tests, and the use of antibiotic therapy. There may have been cases in which the presented symptoms had non-infectious etiology but were not recognized by the doctor. On the other hand, there is also no record of whether the volume of blood collected for the blood cultures was adequate.

In summary, the clinical observation approach has the advantages of reducing laboratory testing and the use of antibiotic therapy without interfering with the prevalence of EOS, when compared to the perinatal risk factors approach. However, a consensus on the concept of EOS is still necessary to enable meta-analyses. Finally, further research is required to determine the best way to systematize serial examinations of newborns and to assess the best symptom prediction method for EOS.

## Conflicts of interest

The authors declare no conflicts of interest.
